# Community readiness to address disparities in access to cancer, palliative and end-of-life care for ethnic minorities

**DOI:** 10.1186/s12889-024-21127-y

**Published:** 2024-12-23

**Authors:** Jodi Emma Wainwright, Erica Jane Cook, Nasreen Ali, Emma Wilkinson, Gurch Randhawa

**Affiliations:** Institute for Health Research, University of Bedfordshire, Luton, UK

**Keywords:** Cancer, Palliative and end-of-life care, Health inequalities, Community readiness, Ethnic minorities

## Abstract

**Background:**

Inequalities in cancer, palliative, and end-of-life care services remain a significant challenge, particularly for ethnic minorities who face systemic barriers such as limited awareness, cultural stigmas, and language differences. These disparities hinder equitable access to essential services and contribute to poorer health outcomes for affected communities. Addressing these challenges requires targeted, culturally sensitive initiatives that promote both awareness and uptake of care. Community readiness is a critical factor in the success of such interventions, as it reflects the willingness and capacity of a community to engage with and support change.

**Methods:**

A mixed-methods approach was used, combining individual interviews and two focus groups with key informants (*N* = 14). This study, conducted in the ethnically and geographically diverse region of Bedfordshire, Luton, and Milton Keynes in southeast England, aimed to assess community readiness to embrace initiatives designed to reduce health inequalities in cancer, palliative, and end-of-life care. The key informants, including faith leaders and professional stakeholders, rated community readiness on five anchored scales: *Knowledge of efforts, Leadership, Knowledge of the issue, Community Climate*, and *Resources*. The focus groups facilitated a discussion of the ratings, providing deeper insights into community dynamics and barriers.

**Results:**

Overall, the community was identified as being at the *pre-planning* stage of readiness to address disparities in cancer, palliative, and end-of-life care for ethnic minorities. Quantitatively, faith and religious leaders assessed readiness at the *vague awareness* stage (mean: 3.88), while professional stakeholders rated it at the *pre-planning* stage (mean: 4.87). Qualitative findings highlighted limited community knowledge, passive leadership with potential for ‘community champions’ to foster openness, a positive climate influenced by younger generations, widespread misconceptions, language barriers, and resource constraints affecting service accessibility.

**Conclusions:**

The Community Readiness Model provides an insight into the community’s position regarding disparities in access to cancer, palliative and end-of-life services. In order to ensure that continuing efforts are successful in addressing existing inequalities rather than exacerbating them, this study emphasises how critical it is to evaluate the readiness of the community in order to avoid widening inequalities in access and use of services.

**Supplementary Information:**

The online version contains supplementary material available at 10.1186/s12889-024-21127-y.

## Introduction

Health inequalities in cancer, palliative, and end-of-life care remain a significant challenge, particularly for socio-economically deprived and ethnic minority communities. In the UK, people in the most deprived areas are more likely to have their cancer diagnosed at a late stage compared to those in the least deprived areas [1]. Furthermore, minority ethnic groups are less likely to be referred for or to access palliative and end-of-life care, and when they do, they are often less satisfied with the care they receive [2, 3]. This inequity is compounded by cultural, social, and systemic barriers, such as lack of awareness, language differences, and mistrust of healthcare services [4].

Palliative and end-of-life care are essential components of cancer care, providing vital support to patients by managing symptoms, enhancing quality of life, and addressing emotional, social, and spiritual needs [5]. Timely access to these services can significantly reduce suffering and improve outcomes for both patients and their families. However, barriers such as delayed cancer diagnosis and inadequate referral pathways often prevent individuals from benefiting from palliative care at the optimal time, leading to poorer quality of life and increased reliance on acute healthcare services at the end of life [6]. Recognising these challenges in the UK, the NHS Long Term Plan [7] prioritises earlier cancer diagnosis, faster referrals, and improved access to personalised, high-quality care. By integrating palliative and end-of-life care within these broader priorities, healthcare systems can address disparities and ensure compassionate, equitable care for all, particularly for underserved populations.

Addressing these disparities requires targeted initiatives that promote equitable access to palliative and end-of-life care, alongside cancer treatment. Community readiness plays a vital role in ensuring these interventions are embraced and effectively implemented. For instance, national initiatives, such as the NHS Core20PLUS5 approach, focus on engaging communities through ‘Community Connectors’ who understand local barriers and work to ensure that underrepresented groups have access to culturally sensitive, person-centred care [8]. By listening to authentic lived experiences and ensuring that services are tailored to the unique needs of these communities, healthcare systems can work towards reducing health inequities in both cancer care and palliative/end-of-life services.

The Community Readiness Model (CRM) [9] is a tool that helps assess a community’s preparedness to address specific issues, such as health disparities or social challenges. It evaluates readiness across stages ranging from *No awareness*, where the issue is barely recognised, to *Community ownership*, where well-supported and sustained efforts are in place (see Supplementary File 1). The CRM considers key dimensions like community knowledge, leadership involvement, attitudes towards change, and available resources. Data is gathered through methods such as interviews and focus groups, enabling a tailored approach to interventions. By aligning actions with a community’s specific stage of readiness, the CRM helps ensure initiatives are effective and sustainable, fostering meaningful progress [10].

The CRM has been successfully applied in other health-related contexts, as documented by Islam et al. [11, 12], these include early intervention programmes aimed at promoting children’s social and emotional health, as well as initiatives addressing obesity within Roma communities. However, this approach has not yet been utilised to explore readiness for addressing disparities in cancer, palliative, and end-of-life care. Additionally, community readiness has only been investigated from a single perspective, for example, to identify and address inequity in end-of-life care in South Asian communities in the UK [13] or to assess American Indian communities’ readiness to address cancer prevention and control programmes in the US [14]. This study, located in a multi-ethnic setting in the UK, seeks to assess the readiness of a diverse community to address disparities in access to cancer, palliative and end-of-life care.

### Context of application

The Denny Review [15], a report that investigated health-related inequalities in Bedfordshire, Luton, and Milton Keynes (BLMK), found a significant gap in patient and community experience of cancer, particularly those from seldom heard populations. One of the outcomes from this review was that BLMK Integrated Care System invested in new ‘Community Connector’ roles to improve access within cancer and end-of-life care pathways for ethnically diverse communities.

The Community Connector role is focused on developing, engaging, and sustaining relationships with the local population through promoting conversations covering topics from cancer symptom recognition, risk factors, access to treatment, care pathway, end-of-life care, good death, loss, and bereavement alongside providing access to, and knowledge about palliative care and the services that a Hospice provides. BLMK Integrated Care System recruited three Community Connectors to work with individuals from the South Asian, Pakistan and Bangladesh; Eastern European; Black African & Caribbean communities who reside within BLMK. Keech Hospice, in Luton, also recruited a Community Connector whose role is to inform and connect the South Asian communities with the hospice.

The ‘Community Readiness Model’ [9] was used as part of a process evaluation to assess whether a ‘Community Connector’ intervention programme was feasible, acceptable to beneficiaries and implementers, and appropriate for the local cultural context within the cancer, palliative, and end-of-life care pathway. The goal of this part of the study was to evaluate the community’s level of readiness to accept the proposed intervention, identifying strengths, gaps, and potential barriers to implementation. By understanding the existing cultural, social, and organisational dynamics within this diverse community, the study sought to ensure that the intervention was contextually relevant and effectively tailored to meet the unique needs and expectations of the populations it aimed to serve.

## Methods

### Study design

The CRM is a versatile mixed-methods framework that combines quantitative readiness scores with qualitative insights from interviews and focus groups. Its adaptability allows it to be tailored to specific local contexts. In this study, we applied the CRM via online individual semi-structured interviews to gather readiness scores and accompanying explanations. These interviews covered five domains, with participants providing detailed explanations for their ratings. Following this, face-to-face focus groups were held to delve deeper into the scores and their rationales. These sessions brought together faith and religious leaders alongside professional stakeholders and community connectors, fostering collaborative discussions about the collective scores and the factors influencing them.

### Study setting

BLMK is a large and diverse geographic region in the Southeast of England, encompassing a population of approximately one million people [16]​. The region is characterised by significant geographical variation, with rural villages, market towns, and urban centres offering contrasting environments. BLMK is also culturally rich, reflecting a mix of ethnicities, languages, and traditions. These differences underline the region’s complexity, requiring tailored approaches to address socio-economic and healthcare inequalities​.

Luton is referred to as ‘super-diverse’, one of the few towns in England where no single ethnic group forms a majority of the population [16]. The population of Luton now has a non-white majority with 55% per cent of the population being non-white. The white ethnic group makes up 45% cent of the population of Luton meaning there is a non-white majority in the town. The Other White category accounting for 11% includes people from Eastern Europe. The Asian group makes up 37%of the population of Luton. The Black ethnic groups make up 10% of the population of Luton [17]. Luton is also linguistically rich, with over 150 languages and dialects spoken across the town. Despite its cultural vibrancy, Luton experiences high levels of socio-economic inequality. Several neighbourhoods are among the 10% most deprived areas in England, with challenges including above-average unemployment rates, child poverty, and limited access to resources in multi-ethnic communities [18]. These disparities emphasise the need for tailored, inclusive policies to address inequalities and foster cohesion.

Bedford Borough is home to residents representing up to 100 ethnic groups and speaking 149 different languages and dialects [16]. The Borough combines urban and rural communities, with two-thirds of the population living in urban centres and the remainder spread across its rural areas. Like other diverse regions in England, Bedford faces challenges associated with socio-economic inequalities, particularly in areas where multi-ethnic communities reside [19]. This includes disparities in access to services and opportunities, which reflect broader patterns of inequality seen both regionally and nationally.

Central Bedfordshire is a predominantly rural area, with over half of its population living in villages and countryside, while the remainder resides in its market towns. The region is generally affluent, characterised by high levels of employment and quality of life, but there are pockets of deprivation that face socio-economic challenges [19]. These disparities highlight the need for targeted support and resources to ensure equitable opportunities and access to services across the area.

Milton Keynes is a diverse area that blends urban and rural communities, including distinct towns and villages. The population is notably young, with 27% of residents aged 19 or under, reflecting its status as one of the fastest-growing cities in the UK [20]. Schools in Milton Keynes reflect its multicultural nature, with students speaking 160 different languages [21]. While the area is dynamic and economically active, the diversity and youthfulness of the population present unique opportunities and challenges in ensuring access to education, services, and community resources.

### Study participants

Faith and community leaders from the Keech Research Partnership Network (KEEP-NET) [22], which encompasses over 80 partner organisations representing the region’s diverse, multi-faith communities, were invited to participate alongside professional stakeholders involved in the main study. A total of fourteen participants were interviewed, including eight local faith and religious leaders, and six professional stakeholders from organisations such as Luton Borough Council, Macmillan Cancer Support, Keech Hospice, and the BLMK Integrated Care Board. These stakeholders contributed their perspectives on community readiness and the barriers and opportunities within the healthcare landscape. More detailed participant information can be found in Table 1 below.

Participants were invited to engage in one of two in-person focus group sessions which took place at different locations within the BLMK area. These sessions provided an opportunity for participants to delve into the results from the individual interviews, using the scores as a starting point for more in-depth discussions. The focus groups allowed stakeholders to explain and contextualise their ratings, offering a richer understanding of the factors influencing community readiness. In addition to faith and community leaders and professional stakeholders, community connectors were an integral part of the focus groups. These connectors helped facilitate discussions by sharing their insights on community engagement and barriers to accessing healthcare services. Their unique perspectives, grounded in local knowledge and experience, helped deepen the conversation around the challenges faced by different communities in engaging with cancer, palliative, and end-of-life care initiatives. The inclusion of community connectors was essential for ensuring that the voices of those directly involved with and trusted by local populations were heard, thus providing a comprehensive understanding of the community’s readiness to embrace change.

**Table 1:** Participants

Participant	Gender	Role*	Sub-group
1	Female	Spiritual Care Lead	1
2	Female	Chaplain	1
3	Female	Chief Executive, health and social care organisation	1
4	Female	Chair of community organisation	1
5	Male	Imam	1
6	Male	Chaplain	1
7	Male	Council of Faiths representative	1
8	Male	Chaplain	1
9	Female	Community support lead	2
10	Female	Senior leader BLMK ICB	2
11	Female	Partnership Manager, charity organisation	2
12	Female	Clinical Director, local hospice	2
13	Female	Public Health Manager	2
14	Male	Project Manager	2

*The roles described here have been generalised so as the participants remain anonymous

### Materials

The Community Readiness Model (CRM) places a community in one of nine possible states, ranging from *No awareness* to *Community ownership* (see Supplementary File 1). Community readiness is assessed across five dimensions: *Community Knowledge of existing efforts*, *Leadership*, *Community Climate*, *Community Knowledge about the issue*, and *Resources*. Each dimension receives a score, allowing for varying levels of readiness across the different areas (see Supplementary File 2). This framework helps identify where a community stands in terms of readiness to address specific issues and guides efforts to move readiness levels forward. These dimensions are key to understanding a community’s capacity to engage with and support interventions aimed at addressing disparities or other challenges.

The ‘gold standard’ [10] protocol requires 4–6 semi-structured interviews per community, each taking approximately 45 min. Interviews are transcribed and scored by two independent scorers using anchored rating scales. However, this process is time and resource intensive. Unlike the standard version where respondents are asked questions with their responses being scored on the anchored rating scales, this study adopts the approach as outlined by Solomon et al. [14] where respondents are instead asked to score a specific area of interest on each of five anchored ranking scales corresponding to the five community readiness dimensions.

The interview responses provided valuable material for the focus group discussions. These 90-minute sessions were guided by the results of the initial interviews, with discussions structured to address the dimensions that scored the lowest first, followed by the next lowest, until all five areas were covered. This approach ensured that the most pressing issues were discussed in detail, allowing for a comprehensive exploration of community readiness. The discussions were audio recorded to capture all insights, while field notes were also taken to provide additional context and support the analysis. This dual approach of audio recording and note-taking enabled a thorough examination of participants’ views, providing a deeper understanding of the community’s perceived readiness and the barriers to engagement with cancer, palliative, and end-of-life care initiatives. The focus groups offered an opportunity for participants to share their perspectives in more depth, facilitating a rich, nuanced understanding of the community’s readiness to tackle these disparities.

### Data analysis

#### Quantitative analysis

Descriptive statistics were used to calculate the mean scores for the CRM interview questions. The scores from individual interviews were aggregated to calculate both domain-specific and overall mean scores, which were reported separately for two sub-groups: (1) faith and community leaders, and (2) professional stakeholders. These results informed the focus group discussions, where the areas with the lowest scores were prioritised. These areas were discussed first, with the aim of identifying potential solutions before addressing the issues with higher scores. This approach ensured that the focus group could tackle the most pressing concerns first, leading to a more targeted strategy for improving community readiness in addressing disparities in cancer, palliative, and end-of-life care.

#### Qualitative analysis

After the interviews and focus groups, data were transcribed and analysed by the main author using a reflexive thematic approach as a framework to organise and interpret the raw data [23]. NVivo 12 software [24] facilitated exploration and coding, enabling systematic identification of key themes. Similarities and differences in individual responses were noted, highlighting nuanced perspectives within and across the groups. The interpretation focused on examining recurring patterns, unique insights, and how these aligned with the dimensions of community readiness.

To ensure the reliability of the coding process, an inter-coder reliability check was conducted with a second coder who independently reviewed a subset of the data. Any discrepancies in coding were discussed and resolved to reach a consensus on theme development, which helped strengthen the rigour of the analysis. Following this, the wider research team reviewed the identified themes, offering further insights to refine the discussion and conclusions. This collaborative review process ensured that the interpretations were contextually grounded, aligned with the study’s objectives, and reflected the diverse perspectives of the participant groups.

## Results

The results in Table 2 reveal both convergence and divergence between quantitative and qualitative findings, highlighting differences in perceptions between faith and religious leaders and professional stakeholders and insights into the readiness of BLMK community to embrace the proposed intervention.

The overall community readiness score, calculated by averaging the five domain scores, was 4.30, placing the BLMK community in the *pre-planning* stage of readiness. However, the mean scores differed between the two sub-groups. Faith and religious leaders (*N* = 8) rated community readiness at 3.88, indicating the communities were at the *vague awareness* stage (stage 3, see Supplementary File 1). This score suggests limited awareness of existing efforts and the issue itself, coupled with passive or minimal leadership involvement.

In contrast, professional stakeholders (*N* = 6) rated the communities higher, with a mean score of 4.87, indicating they perceived the communities to be in the *pre-planning* stage (stage 4). This stage suggests some recognition of the issue and emerging leadership support, though concrete planning efforts may still be limited. These discrepancies highlight differences in perspectives between the two groups, which may stem from their varying roles, experiences, and levels of engagement within the community.

**Table 2:** Quantitative data

	Participant	Community knowledge of efforts	Leadership	Community climate	Community knowledge of the issue	Resources	Overall Readiness
Group 1	1	2	4	7	6	5	4.80
	2	4	4	3	3	4	3.60
	3	4	5	4	2	4	3.80
	4	2	7	6	4	3	4.40
	5	2	3	4	3	3	3.00
	6	4	3	4	5	3	3.80
	7	2	5	5	5	2	3.80
	8	3	4	5	4	3	3.80
Group 2	9	3	4	4	2	5	3.60
	10	3	6	9	3	6	5.40
	11	2	6	5	6	6	5.00
	12	5	5	6	4	7	5.40
	13	4	5	6	3	7	5.00
	14	5	6	5	3	5	4.80
Total	Mean	3.21	4.79	5.21	3.79	4.50	4.30
	SD	1.12	1.19	1.53	1.31	1.61	0.76
Group 1	Mean	2.88	4.38	4.75	4.00	3.38	3.88
	SD	0.99	1.30	1.28	1.31	0.92	0.53
Group 2	Mean	3.67	5.33	5.83	3.50	6.00	4.87
	SD	1.21	0.82	1.72	1.38	0.89	0.67

The results, presented in more detail below, further illustrate these findings. Bold highlights indicate progression between readiness stages, emphasising how perceptions of the community’s preparedness vary across domains and stakeholder groups. This nuanced understanding of community readiness provides valuable insights for tailoring interventions to address disparities in cancer, palliative, and end-of-life care access effectively.

### Community knowledge of efforts

The overall mean score for community knowledge of efforts was 3.21 (SD = 0.56), indicating a moderate level of awareness among the broader community. When examining specific groups, faith and religious leaders reported a mean score of 2.88 (SD = 0.99), suggesting a relatively lower level of knowledge compared to other stakeholders. In contrast, professional stakeholders demonstrated a higher level of awareness, with a mean score of 3.67 (SD = 1.21). This disparity highlights potential differences in access to or engagement with information about these efforts between faith leaders and professional stakeholders. These findings suggest the need for targeted strategies to bridge the knowledge gap and ensure equitable dissemination of information across all community groups.

Overall readiness: Stage 3 – At least **some** community members **have heard of local efforts, but little else**.

Participants reported that while efforts are being made to improve access to cancer, palliative, and end-of-life care, community members’ knowledge of these efforts is still limited. As Participant 4 (Chair of community organisation) explained, “*few people are aware*.” This suggests that despite ongoing initiatives, many community members remain uninformed, possibly due to ineffective communication, cultural barriers, or lack of targeted outreach. Ensuring broader awareness and engagement is crucial to improving access to these services.

The community connector intervention is still in the early stages of a three-year project, with efforts to build connections within communities developing but ongoing. Participant 13 (Public Health Manager) noted, “*Some community groups have been very engaged and interactive*, *however*, *there are still many who are not*.” This reflects the varying levels of engagement, with some groups actively participating while others remain less involved. Overcoming this gap will be crucial as the project progresses, requiring targeted outreach to ensure broader community involvement.

General awareness is improving and opportunities for two-way communication are increasing thus conversations are increasing in number but remain an issue in some parts of the community. Local events are beginning to raise awareness of efforts and of the issue, “*an understanding of what end of life care is about*, *whether that’s services in the community or whether that’s services in the hospice. But also*, *we’ve started to understand more about what different communities require of us*, *and we’ve started to make adjustments to our services*” (Participant 12, Clinical Director).

### Leadership

The overall mean score was 4.79 (SD = 1.19), reflecting a relatively high level of agreement or positivity across all respondents. When analysed by specific groups, faith and religious leaders reported a mean score of 4.38 (SD = 1.30), indicating slightly lower levels compared to the overall mean, but still within a range suggesting general positivity. On the other hand, professional stakeholders reported a notably higher mean score of 5.33 (SD = 0.82), indicating stronger agreement or more positive perceptions within this group. These findings reveal a difference between the perspectives of faith leaders and professional stakeholders, which could point to variations in priorities, experiences, or access to resources. Addressing this discrepancy may require tailored approaches to ensure alignment and mutual understanding across all stakeholder groups.

Overall readiness: Stage 4 - At least some of the leadership believes that this issue **is** a concern in the community **and that some type of effort is needed to address it**. Although some may be at least passively supportive of current efforts, **only a few may be participating in developing, improving or implementing efforts**.

Participants in both focus groups mentioned collaboration, there is a recognition of needs and relations between community members and leaders are improving, however, there was an acknowledgement that some faith and religious leaders are from an older generation and may need more persuasion to open up and discuss the issue.

The notion of ‘community champions’ could have a positive influence with local religious and faith leaders taking on this role and providing good examples of those who access care services that are available. Positive stories will encourage openness and opportunities to talk. The second focus group talked about celebrities who talk about their experiences, “*if EastEnders has an episode on something and all of a sudden you get all these calls and people coming forward*” (Participant 10, Senior leader BLMK ICB), this is encouraging openness and normalising the issue.

Some participants expressed their concern that there is a lot of ‘talking’ and not much ‘doing’, there is a need for the community to know the outcomes of current and future initiatives, “*feedback is key to building relationships”* (Participant 9, Community support lead).

There is still a need for training for healthcare professionals to understand cultural differences, as this will improve care when ethnic minorities access services. As Participant 3 (Chief Executive, health and social care organisation) expressed “*it’s often the same people around the same tables*”. This reflects the issue that decision-making often lacks diverse voices, which can limit the understanding of the community’s specific needs. Including a broader range of perspectives is essential for creating more culturally competent and effective healthcare.

### Community climate

The overall mean score for community climate was 5.21 (SD = 1.53), reflecting a generally positive perception among respondents. Faith and religious leaders reported a mean score of 4.75 (SD = 1.28), indicating moderately positive views, but slightly below the overall average. In contrast, professional stakeholders reported a higher mean score of 5.83 (SD = 1.72), suggesting a more favourable assessment of the community climate. This disparity points to differences in perspectives, possibly influenced by varying levels of engagement, resource access, or experiences within the community. These results highlight the importance of fostering collaboration and addressing the unique needs of each group to ensure a cohesive and inclusive community climate.

Overall readiness: Stage 5 - At least **some** community members are **participating in developing, improving, or implementing efforts**, possibly attending group meetings that are working toward these efforts.

Participants described how educating the younger generations will help the community climate as a whole. By improving understanding from a young age, there will be less misconceptions and more openness talking about the issues around cancer, palliative and end-of-life care. There are, however, difficulties in getting some cultures to change, “*if a culture hasn’t spoken about death for over 2000 years*, *it’s difficult to get them to change overnight*, *it takes time*” (Participant 2, Chaplain).

Attempts to improve knowledge about cancer, palliative, and end-of-life care often stem from external institutions, such as hospitals or hospices, rather than emerging organically from within the communities themselves. As Participant 1 (Spiritual Care Lead) explained “*I don’t think it’s something that has been requested*”.

Ethnic minorities are the focus of many local initiatives, but it was noted that other groups, such as the LGBTQI community, also face barriers to access and inclusion. While efforts for ethnic minorities have made progress, there is a need to improve awareness and access for LGBTQI individuals. Taking an intersectional approach can ensure that all marginalised groups are represented and supported through tailored programmes and equitable access to resources.

### Community knowledge of the issue

The overall mean score for community knowledge of the issue was 3.79 (SD = 1.31), indicating a moderate level of awareness across respondents. Faith and religious leaders reported a mean score of 4.00 (SD = 1.31), suggesting a slightly higher-than-average understanding of the issue within this group. In contrast, professional stakeholders had a mean score of 3.50 (SD = 1.38), reflecting a slightly lower level of knowledge compared to faith leaders and the overall mean. This variation may highlight differences in exposure to or prioritisation of the issue among these groups. While faith and religious leaders might have a closer connection to community-level concerns, professional stakeholders may require additional engagement or information dissemination to ensure a shared understanding of the issue. These findings underscore the importance of customised communication strategies to bridge gaps in knowledge and foster collaboration among all stakeholder groups.

Overall readiness: Stage 3 - **At least some** community members have **heard of the issue, but little else**. Among some community members, there **may** be misconceptions about the issue. Community members **may be somewhat aware that the issue occurs locally**.

Participant 14 (Project manager) explained, “*I think sometimes we underestimate how much community members know about the issue*,” suggesting that there is often an assumption that ethnic minority communities are less informed about healthcare issues like cancer and end-of-life care. However, there was consensus across both groups that misconceptions were a real problem, there are misconceptions regarding many aspects including ethnicity and age. Different faiths and different cultures have different understandings, there are misconceptions about what treatments are available, community members often believe that they don’t have a choice, this is an area that can be improved.

Language is also a barrier that was identified by the participants, information needs to be more accessible in terms of languages spoken and understood by the community members as well as terminology – simplified for ease of understanding. ‘Phase of life’ is a term that some participants are using to replace ‘end of life’ as it translates better and is better understood by many in the community, “*it’s about being mindful of language and what it means to different people*” (Participant 9, Community support lead).

### Resources

The overall mean score for resources was 4.50 (SD = 1.61), indicating a moderate perception of resource availability across respondents, but significant disparities emerged between groups. Faith and religious leaders reported a lower mean score of 3.38 (SD = 0.92), suggesting limited access to or adequacy of resources in their contexts. In contrast, professional stakeholders reported a much higher mean score of 6.00 (SD = 0.89), reflecting strong confidence in resource sufficiency within their professional environments. This disparity highlights potential inequities, with faith and religious leaders likely facing challenges such as limited funding or infrastructure, while professional stakeholders benefit from greater institutional support. These findings underline the importance of addressing resource gaps and ensuring equitable support to empower all stakeholders in their efforts.

Overall readiness: Stage 4 - There are some resources identified that could be used for further efforts. **Some community members or leaders have looked into or are looking into using these resources** to address the issue.

Participants were reminded that resources could include a range of essentials necessary for community initiatives, such as funding, trained personnel, transportation, and educational materials. These resources are critical for ensuring that individuals can access services, especially in underserved areas, and for facilitating effective outreach and support. A comprehensive approach to resource allocation is crucial for the success of efforts to improve access to cancer, palliative, and end-of-life care.

Professional stakeholders reported higher scores than the religious and faith leaders who participated. This difference could stem from a lack of awareness among faith leaders about available resources. For example, Participant 7 (Council of Faiths representative) stated, “*I don’t really know whether there is any funding*,” and Participant 8 (Chaplain) mentioned, “*I’m not really aware of what resources are available*.” These comments suggest that faith leaders may have different access to information about funding or resources than professional stakeholders, highlighting the need for better communication and collaboration between healthcare professionals and faith communities.

Creativity was mentioned as a positive element of current resources, local events that have taken place demonstrate a holistic approach to dealing with the issue. The second focus group talked about how the local workforce has begun to represent local communities better, “*we have seen our workforce change for the better as well so the two have gone hand in hand so as we have done more community engagement*, *I think talk more about our services we have recruited from a more diverse community*” (Participant 12, Clinical Director).

Participants also mentioned time as a strength and a threat, “*it’s not the money it’s the time*” (Participant 11, Partnership Manager). There is an acknowledgement that improving access to services will take time, building on trust. However, a person’s time is limited. The community connectors are part-time yet each feels they could do so much more if they had more working hours.

The quantitative scores provided a broad indication of readiness, while qualitative insights enriched this understanding by shedding light on specific challenges and opportunities. The discrepancy between the subgroups—particularly in resource perception—points to the need for improved communication and alignment of expectations among stakeholders. Together, these findings underscore the importance of addressing barriers such as misconceptions, leadership engagement, and resource allocation to advance the community’s readiness for the proposed intervention.

## Discussion

This study contributes to the growing body of research on community readiness and health disparities by applying the Community Readiness Model (CRM) to explore disparities in cancer, palliative, and end-of-life care for ethnic minority communities in a multi-ethnic setting. By identifying readiness levels at both local and subgroup levels, it highlights challenges and opportunities for improving equity in healthcare access and delivery. The findings also underscore the importance of tailored, culturally appropriate interventions to address systemic barriers.

The overall readiness score reflected in the diverse community was the *pre-planning* stage, suggesting that while there is some awareness of disparities, concrete strategies and resources remain underdeveloped. This aligns with findings from previous CRM applications in diverse communities, such as Moss et al. [13], who reported that South Asian communities in the UK also fell within the *pre-planning* stage when assessing end-of-life care readiness. Similarly, international studies, such as those by Ahmed et al. [3], noted that limited awareness, stigma, and cultural norms frequently hindered minority populations’ access to palliative care globally.

The differences in readiness perceptions between faith and community leaders (3.88, *vague awareness*) and professional stakeholders (4.87, *pre-planning*) reveal distinct viewpoints on the barriers to equitable care. Faith and community leaders highlighted gaps in public knowledge and cultural stigma, particularly around discussing end-of-life care. Professional stakeholders, however, perceived some progress, likely reflecting their proximity to ongoing initiatives. This disparity suggests a need for greater collaboration and communication across these groups to ensure that interventions are informed by diverse perspectives. By fostering more collaborative partnership working, initiatives can be better aligned with the unique needs of all minority groups, ensuring that solutions are inclusive and comprehensive. Such partnerships can facilitate the sharing of knowledge, resources, and expertise, ultimately leading to more effective and equitable outcomes for the community as a whole.

National and international literature reinforces the importance of addressing ethnic disparities in healthcare access and outcomes. The Denny Review [15] and the NHS Long-Term Plan [7] both identify ethnic disparities in healthcare access and outcomes as critical challenges, particularly in cancer and palliative care. Similarly, Koffman and Higginson’s [25, 26] studies on stigma in end-of-life care among minority groups emphasise the role of trust and culturally competent care in improving engagement. Other evidence highlights the effectiveness of community-driven approaches in addressing health inequities, demonstrating that readiness assessments can inform the design of impactful interventions [27, 28].

Qualitative data from this study emphasized the importance of educational campaigns and community engagement in raising awareness and reducing stigma, with Community Connectors facilitating discussions and fostering trust. This model mirrors successful international initiatives. In Southeastern Australia, community connectors bridge cultural and organisational gaps to engage hard-to-reach populations [29]. Western Australia’s Compassionate Communities Connectors expand support networks for individuals with advanced illnesses [30], while in New Zealand, connectors help people access welfare and social services, complementing broader support frameworks [31]. These models highlight the efficacy of local, community-driven solutions to address healthcare disparities and improve access to essential services.

Despite the challenges, the optimism expressed by participants about the potential for progress is encouraging. The concept of a 'butterfly effect,' where small changes in awareness could lead to significant outcomes, resonates with broader public health literature, which suggests that incremental improvements in education and engagement can lead to long-term cultural shifts [32, 33].

This study also highlights the CRM’s utility as a framework for evaluating and guiding interventions in complex, multi-ethnic contexts. By identifying specific readiness dimensions—such as knowledge of efforts and knowledge of the issue —where improvements are needed, the CRM provides actionable insights for policymakers and practitioners. For example, investing in culturally tailored educational initiatives, improving service accessibility, and fostering collaborations between community leaders and healthcare professionals could help advance readiness to higher stages.

### Strengths and limitations

The Community Readiness Model (CRM) has been successfully applied in various health contexts to assess readiness for addressing health disparities. Whereas this study represents the first use of the CRM to explore disparities in cancer, palliative, and end-of-life care in a multi-ethnic community in the UK. Previous applications of the CRM have often focused on specific populations, such as identifying inequities in end-of-life care among South Asian communities in the UK [13] or assessing readiness for cancer prevention and control in American Indian communities in the US [14]. Unlike these studies, which examined readiness from a single perspective, this research captures the perspectives of diverse groups, including faith leaders, community connectors, and professional stakeholders.

By situating the study in a multi-ethnic UK community, it provides unique insights into the barriers and opportunities for addressing healthcare disparities across varied cultural and socio-economic contexts. Furthermore, the integration of both qualitative and quantitative data through interviews and focus groups enriches the findings and offers a nuanced understanding of community readiness. This novel application of the CRM broadens its scope and demonstrates its utility in tackling complex, multi-dimensional health inequalities, thereby setting the groundwork for more inclusive, community-informed interventions in cancer, palliative, and end-of-life care.

This study has several limitations that should be considered when interpreting the findings. First, the relatively small sample size may limit the generalisability of the results. With only fourteen participants the findings may not fully capture the diversity of perspectives across all segments of the BLMK ethnic minority communities. Although the selected participants provided valuable insights, a larger sample might have offered a broader understanding of community readiness and identified additional nuances within subgroups.

Second, while the data collection process was systematic, member checking was not conducted. Member checking, which involves validating findings with participants to confirm the accuracy of interpretations, can help ensure that the analysis accurately reflects participants’ perspectives. The absence of member checking in this study may mean that some interpretations were not fully validated by those providing the data, potentially affecting the credibility of the findings.

Additionally, the study’s questions were adapted from the Community Readiness Model without pilot testing in the specific context of cancer, palliative, and end-of-life care for ethnic minorities. Piloting the questions with a sample representative of the study population could have helped refine questions to ensure clarity and relevance, thereby improving the reliability and validity of the responses. Without this step, there is a risk that some questions may not have fully captured participants’ views or may have led to misinterpretation.

These limitations highlight areas for potential refinement in future research, such as expanding the sample size to capture a wider range of perspectives across BLMK’s diverse communities. Incorporating member checking could strengthen the alignment between participants’ views and the study’s interpretations, while pilot testing the questionnaire in similar settings would ensure that the questions are optimally tailored to the context of cancer, palliative, and end-of-life care for ethnic minorities. By addressing these aspects, future studies can build on the current findings, further enhancing the reliability and depth of insights into community readiness in this vital area.

## Conclusion

In conclusion, this research highlights the critical need to understand and address community-specific barriers to healthcare access, using the Community Readiness Model (CRM) as a framework for evaluating the readiness of diverse communities to address health disparities. While the findings are centred in the BLMK region, the lessons and insights drawn are applicable globally, providing a model for similar communities. Future research should build on this work by exploring the long-term effects of readiness-based interventions, evaluating their scalability, and adapting the CRM to address the diverse healthcare needs of different populations.

## Electronic supplementary material

Below is the link to the electronic supplementary material.

Supplementary Material 1

## Data Availability

The datasets used and analysed during the current study are available from the corresponding author upon reasonable request.

## Abbreviations

BLMK: Bedford, Luton and Milton Keynes
CRM: Community Readiness Model
ICS: Integrated Care System
NHS: National Health Service
UK: United Kingdom
US: United States
