# Supplementary material for: Community readiness to address disparities in access to cancer, palliative and end-of-life care for ethnic minorities
Source: BMC Public Health. 2024 Dec 23;24:3566. doi: 10.1186/s12889-024-21127-y (PMC11668017; doi:10.1186/s12889-024-21127-y)
Supplement: Supplementary file 1 — Supplementary Material 1 [file 12889_2024_21127_MOESM1_ESM.pdf]

Supplementary Material 1: Key features of each community readiness model stage.

Stage		Key features
**1**	No awareness	• Community has **no knowledge** about local efforts addressing the issue.<br>• Leadership believes that the issue **is not really much** of a concern.<br>• The community believes that the **issue is not a concern.**<br>• Community members have **no knowledge** about the issue.<br>• There are **no resources** available for dealing with issue.
**2**	Denial/Resistance	• Leadership and community members believe that this issue **is not** a concern in their community or they think it **can't or shouldn't be addressed.**<br>• Community members have misconceptions or **incorrect knowledge** about current efforts.<br>• **Only a few** community members have **knowledge** about the issue, and there may be many misconceptions among community members about the issue.<br>• Community members and/or leaders **do not support using available resources** to address this issue.
**3**	Vague awareness	• **A few** community members have heard about local efforts, **but know little about them.**<br>• Leadership and community **members believe this issue may be a concern in the community**. They show no immediate motivation to act.<br>• Community members have only **vague knowledge** about the issue (e.g., they have some awareness that the issue can be problem and why it may occur).<br>• There are **limited resources** (such as a community room) identified that could be used for further efforts to address the issue.
**4**	Pre-planning	• **Some** community members have at least heard about local efforts, but **know little about them**.<br>• Leadership and community members **acknowledge that this issue is a concern** in the community and that something has to be done to address it.<br>• Community members have **limited knowledge** about the issue.<br>• There are **limited resources** that could be used for further efforts to address the issue.
**5**	Preparation	• **Most** community members have at least heard about local efforts.<br>• Leadership is **actively supportive of continuing or improving current efforts** or in developing new efforts<br>• The attitude in the community is "**We are concerned** about this and **we want to do** something about it".<br>• Community members have **basic knowledge** about causes, consequences, signs and symptoms.<br>• There are **some resources** identified that could be used for further efforts to address the issue; community members or leaders are actively working to secure these resources.

**6**	Initiation	• **Most** community members have at least **basic** knowledge of local efforts.<br>• Leadership **plays a key role** in planning, developing and/or implementing new, modified, or increased efforts.<br>• The attitude in the community is "**This is our responsibility"**, and some community members are involved in addressing the issue.<br>• Community members have **basic knowledge** about the issue and are **aware that the issue occurs locally.**<br>• **Resources have been obtained** and/or allocated to support further efforts to address this issue.
**7**	Stabilisation	• **Most** community members have **more than basic knowledge** of local efforts, including names and purposes of specific efforts, target audiences, and other specific information.<br>• Leadership is **actively involved in ensuring or improving the long-term viability** of the efforts to address this issue.<br>• The attitude in the community is 'We **have taken** responsibility". There is **ongoing** community involvement in addressing the issue.<br>• Community members have **more than basic knowledge** about the issue.<br>• A considerable part of allocated resources for efforts are from sources that are expected to provide **continuous support**.
**8**	Expansion/ Confirmation	• **Most** community members have **considerable** knowledge of local efforts, including the level of program effectiveness.<br>• Leadership **plays a key role in expanding and improving efforts**.<br>• The majority of the community **strongly** supports efforts or the need for efforts. Participation level is high.<br>• Community members have **more than basic** knowledge about the issue and have **significant** knowledge about **local prevalence** and local consequences.<br>• A considerable part of allocated resources are expected to provide **continuous** support. Community members are looking into **additional** support to implement new efforts.
**9**	Community Ownership	• **Most** community members have **considerable and detailed** knowledge of local efforts,<br>• Leadership is **continually reviewing evaluation** results of the efforts and is modifying financial support accordingly.<br>• **Most** major segments of the community are highly supportive and actively involved.<br>• Community members have **detailed** knowledge about the issue and have **significant** knowledge about **local prevalence** and local consequences.<br>• Diversified resources and funds are secured, and efforts are expected to be ongoing.

Supplementary Material 2: Anchored rating scales for scoring each dimension

Question A: Knowledge of efforts:

What is your assessment of how knowledgeable members of the community are about the local initiatives/practices to improve access to cancer, palliative and end-of-life care in a way that means services can prioritise what matters most to patients and to their loved ones.

**Level**	**Description**
1	Community members have **no knowledge** about local efforts addressing the issue.
2	**Only a few** community members have **any knowledge** about local efforts addressing the issue. Community members may have **misconceptions or incorrect knowledge** about local efforts (e.g., their purpose or who they are for).
3	At least **some** community members **have heard of local efforts, but little else.**
4	At least some community members have heard of local efforts and **are familiar with the purpose of the efforts.**
5	At least some community members have heard of local efforts, are familiar with the purpose of the efforts, **who the efforts are for, and how the efforts work.**
6	**Many** community members have heard of local efforts and are familiar with the purpose of the effort. At least some community members know who the efforts are for and how the efforts work.
7	Many community members have heard of local efforts, are familiar with the purpose of the effort, **who the efforts are for, and how the efforts work. At least a few community members know the effectiveness of local efforts.**
8	**Most** community members have heard of local efforts and are familiar with the purpose of the effort. **Many** community members know who the efforts are for and how the efforts work. **Some** community members know the effectiveness of local efforts.
9	Most community members have **extensive** knowledge about local efforts, **knowing the purpose, who the efforts are for and how the efforts work. Many** community members know the effectiveness of the local efforts.

Question B: Leadership:

This question is about local leadership – how those people who make key decisions about health and faith consider access to cancer, palliative and end-of-life care.

**Level**	**Description**
1	Leadership believes that the issue **is not** a concern.
2	Leadership believes that this issue may be a concern in this community, but **doesn't think it can or should be addressed.**
3	At least some of the leadership **believes that this issue may be a concern in this community**. It may not be seen as a priority. They show no immediate motivation to act.
4	At least some of the leadership believes that this issue **is** a concern in the community **and that some type of effort is needed to address it.** Although some may be at least passively supportive of current efforts, **only a few may be participating in developing, improving or implementing efforts**.
5	**At least some of the leadership is participating in developing, improving, or implementing efforts**, possibly being a member of a group that is working toward these efforts or being supportive of allocating resources to these efforts.
6	**At least some of the leadership plays a key role** in participating in current efforts and in developing, improving, and/or implementing efforts, possibly in **leading** groups or **speaking out publicly** in favour of the efforts, and/or as other types of **driving forces**.
7	At least some of the leadership plays a key role in **ensuring or improving the long- term viability** of the efforts to address this issue, for example by allocating long-term funding.
8	At least some of the leadership plays a key role in **expanding and improving efforts**, through **evaluating** and **modifying** efforts**, seeking new resources**, and/or helping develop and implement new efforts.
9	At least some of the leadership is continually **reviewing evaluation results** of the efforts and is **modifying financial support accordingly.**

Question C: Community climate:

This question is about “Community climate”. This is a difficult dimension to be specific about – it’s around how much you think this is an issue that would resonate with local communities.

Level	Description
1	Community members believe that the issue is **not** a concern.
2	Community members believe that this issue may be a concern in this community, **but don’t think it can or should be addressed.**
3	Some community members **believe that this issue may be a concern in the community, but it is not seen as a priority**. They show no motivation to act.
4	Some community members believe that this issue **is** a concern in the community and that **some type of effort is needed to address it**. Although some may be at least passively supportive of efforts, **only a few may be participating in developing, improving or implementing efforts.**
5	At least **some** community members are **participating in developing, improving, or implementing efforts**, possibly attending group meetings that are working toward these efforts.
6	At least **some** community members **play a key role in** developing, improving, and/or implementing efforts, possibly being members of groups or speaking out publicly in favor of efforts, and/or as other types **of driving forces.**
7	At least some community members play a key role in **ensuring or improving the long-term viability** of efforts (e.g., example: supporting a tax increase). The attitude in the community is —” We have taken responsibility”.
8	The **majority** of the community **strongly** supports efforts or the need for efforts. **Participation level is high**. —” We need to continue our efforts and make sure what we are doing is effective.”
9	The majority of the community are **highly supportive** of efforts to address the issue. **Community members demand accountability.**

Question D: Community knowledge:

This question refers to members of the community, local people including those who may need to plan end-of-life care.

**Level**	**Description**
1	Community members have **no** knowledge about the issue.
2	**Only a few** community members have **any knowledge** about the issue. Among **many** community members, there are **misconceptions** about the issue, (e.g., how and where it occurs, why it needs addressing, whether it occurs locally).
3	**At least some** community members have **heard of the issue, but little else.** Among **some** community members, there **may be** misconceptions about the issue. Community members **may be somewhat aware that the issue occurs locally.**
4	At least some community members **know a little about causes, consequences, signs and symptoms.** At least some community members **are** aware that the issue occurs locally.
5	At least some community members know **some** about causes, consequences, signs and symptoms. At least some community members are aware that the issue occurs locally.
6	At least some community members know some about causes, consequences, signs and symptoms. At least some community members have some knowledge about **how much it occurs locally** and **its effect on the community.**
7	At least some community members **know a lot** about causes, consequences, signs and symptoms. At least some community members have some knowledge about how much it occurs locally and its effect on the community.
8	**Most** community members know a lot about causes, consequences, signs and symptoms. At least some community members have **a lot** of knowledge about how much it occurs locally, its effect on the community, and how to address it locally.
9	Most community members have **detailed** knowledge about the issue, knowing **detailed information** about causes, consequences, signs and symptoms. **Most** community members have **detailed** knowledge about how much it occurs locally, its effect on the community, and how to address it locally.

Question E: Resources:

This question relates to resources available to implement local access to cancer, palliative and end-of-life care. It is concerned with the capacity to develop/improve services beyond those currently in place.

**Level**	**Description**
1	There are **no** resources available for (further) efforts.
2	There are very **limited** resources available that could be used for further efforts. There is no action to allocate these resources to this issue. Funding for any current efforts is not stable or continuing.
3	There are **some** resources that could be used for further efforts. There is **little** or no action to allocate these resources to this issue.
4	There are some resources identified that could be used for further efforts. **Some community members or leaders have looked into or are looking into using these resources** to address the issue.
5	There are some resources identified that could be used for further efforts to address the issue. Some community members or leaders are **actively working to secure these resources**; for example, they may be **soliciting donations, writing grant proposals, or seeking volunteers.**
6	**New resources** have been **obtained and/or allocated** to support further efforts to address this issue.
7	A **considerable part** of allocated resources for efforts **are from sources that are expected to provide stable or continuing support.**
8	A considerable part of allocated resources for efforts are from sources that are expected to provide continuous support. **Community members are looking into additional support to implement new efforts.**
9	**Diversified resources and funds are secured, and efforts are expected to be ongoing. There is additional support for new efforts.**
